# Indirect Osteoplasty

**Published:** 1869-08

**Authors:** 


### Indirect Osteoplasty.

Prof. Billroth, in a contribution on the results of operative proceedings for favoring the regeneration of bone from periosteum, states that, in diseased conditions of the full grown hollow bones of adult subjects, the periosteum resembles that found in early life, in being lined by a layer of cells which may be converted into irregular and luxuriant masses of osseous tissue. This progress is observed in cases of ostitis and acute periostitis, particularly in syphilitic subjects, in whom there is a great tendency to the formation of osteophytic growths. The sanguine expectations with which periosteal osteoplastic operations were undertaken have not, however, been fulfilled. In the first place, the surgeon has no power to limit or control the abundant formation of bone-cells from diseased periosteum ; and, again, the newly formed bone after a time contracts like other regenerated connective tissue, and finally wholly dissappears. Artificial osteogenesis may be produced in children, after resection, when the wound heals by primary intention; but it fails in adults, and when the wound remains open for some time, with profuse suppuration. Rhinoplastic operations, in which flaps of periosteum had been detached from the frontal bone, though followed by formation of osseous tissue in the new nose, were ultimately unsatisfactory in their results, in consequence of the absorption of this tissue, and of the formation of an immoveable and tense cicatrix on the forehead. The transplanting of periosteal flaps in operations for cleft palate is not approved of by Billroth, in consequence of the great difficulty of the proceeding, and of its slight utility. In cases of necrosis of the gums from phosphorus-poisoning, success has frequently attended periosteal resection when the formation of new bone was not prevented through profuse suppuration. The formation of a perfect maxillary bone must not be expected : in regeneration of the upper jaw, the antrum is lost, the bone itself is flattened, and the cheek sunken. In resection of joints, periosteal operations, in consequence of the softened and relaxed condition of the membrane, have not resulted in marked success, except in a few cases, in which they were practiced on young subjects, and where healing was perfected without severe symptoms.—*Allgemeine Wiener Medizinische Zeitung*, No. 49, 1868.—*New Orleans Medical Journal.*

